# Hospital and Institutional News

**Published:** 1914-11-21

**Authors:** 


### HOSPITAL AND INSTITUTIONAL NEWS.

### THE FIRST MEDICAL VICTORIA CROSS.

MEDICAL men in this country and with the Forces cannot fail to note with pride that, in the first list of nine names which have been awarded the Victoria Cross for valour, is included that of a member of the Royal Army Medical Corps, Captain Harry Sherwood Ranken, R.A.M.C. The Supplement to the *London Gazette*, issued on Monday last, which announces the awards of the Victoria Cross to nine members of the British Expeditionary Force, contains the following official intimation of the "conspicuous bravery" for which Captain Harry Ranken has been honoured: "Captain Harry Sherwood Ranken, Royal Army Medical Corps. For tending wounded in the trenches under rifle and shrapnel fire at Hautvesnes on September 19, and on September 20 continuing to attend to wounded after his thigh and leg had been shattered." It is interesting to add that of the nine men who have been awarded the V.C. up to date, five are officers and four non-commissioned officers, while two of the latter, belonging to the already distinguished L Battery, Royal Horse Artillery, have since been awarded commissions. We have also regretfully to add that two of the recipients, including Captain Ranken, have since died of their wounds.

### THE MEANING OF MEDICAL VALOUR.

OUR readers are placed in an exceptionally advantageous position to realise the relation of members of the Royal Army Medical Corps to the display of valour in the field from the pregnant remarks on this subject which were contained in the article which we published on November 7 from a Medical Officer at the Front. Taking as his text the regimental medical officer's duties, our contributor pointed out, in the light of the practical experience which he and his colleagues have had so abundantly since the war began, that it was impossible for the medical officer in modern warfare "to be in more than a small fraction of the actual firing-line at any one time." The result of this in practice, our contributor added, is that "if he intends to succour the wounded actually where they fall he must travel long distances up and down a wide front, exposing himself necessarily to the enemy's fire in so doing." Reasons were then given why it was not desirable from the strictly military point of view to do this, and it was argued that the wounded were "far safer lying where they fall—provided someone will apply the first field-dressing which every soldier carries—than being taken away in daylight while the action is proceeding." The writer concluded by pointing out that this lesson had been driven home by the additional wounds which the wounded had suffered during attempts at removal, and by the higher proportion of casualties among the regimental medical officers and stretcher-bearers. Till this lesson was learnt, we were told, a misguided gallantry and the hope of winning the V.C. had proved a very real temptation to the Royal Army Medical Corps. The moral of this is that the commanding officers whose troops have suffered through the drain on the medical *personnel* are now in a position to recommend for the V.C. and similar distinctions only those who have shown that highest form of valour, in which common sense and self-control have their due share. We can therefore acclaim the new V.C.s with sincerity, knowing that the decoration is likely, from the circumstances of modern warfare, to be the reward of a type of valour less reckless and more genuine than has perhaps been possible in the past.

### THE QUEEN'S VISIT TO THE AMERICAN WOMEN'S WAR HOSPITAL.

OLDWAY HOSPITAL, Paignton, established by the American Women's War Committee, was visited by the Queen on Thursday, last week. It was intended to have been a private visit, but a large crowd assembled and the greatest enthusiasm was displayed. The Queen was met at the station by Colonel Gunning, commandant at the hospital, and Mr. and Mrs. Paris Singer. Her visit at "Oldway" extended to nearly two hours. She spoke words of sympathy and encouragement to each of the wounded soldiers, many of whom had the pieces of shrapnel and bullets to show to Her Majesty. The Queen noticed with interest the tattoo work on the arm of one of the men representing the King and herself, which she ascertained had been done during the Royal visit to India, where the man was stationed at that time. The Queen was very much pleased with the equipment of the institution, and it is perhaps worthy of remark in connection with her visit that the special train in which she travelled left Paddington at eleven, and left Paignton for the return journey at 4 P.M. Even a few years ago such a hospital visit in the time would not have been possible.

### THE FIVE POINTS OF A SUCCESSFUL APPEAL.

THE letters and comments which have been appearing of late in THE HOSPITAL on the art of appealing in time of war have brought us several suggestions, and among them the following concrete proposal. "Could not some of the most experienced and successful secretaries," writes an institutional manager, "lay down, say, five points to be borne in mind in order to obtain successful results, or publish in THE HOSPITAL an analysis of some good appeals?" The suggestion is surely worth acting upon, for such inter-co-operation is the essence of the voluntary system; and as the writers can remain, if they prefer it, anonymous there need be no fear, in this case, that one institution will be injured if the secrets of the art on which its appeals are based is made known. The appeal which is graphic, terse, pointed, and moving—ending, as someone once remarked, "with a sob or a smile"—is indeed of permanent suggestive value; but often no record is kept, and we, therefore, invite hospital secretaries to send us the best-drafted appeals which they have observed. There should be no difficulty about this, since the obvious feature of a good appeal is that it clings in the memory; and a collection of first-rate examples would be of lasting interest and value. Will our readers, therefore, send us any appeals which have impressed them, or where the manuscript has perished, the points, at least, which they most vividly recall?

### A DISPENSING DISASTER AT BETHLEM HOSPITAL.

Dr. F. J. Waldo, the Southwark Coroner, held an inquest last week concerning the death of five patients at Bethlem Hospital from the effects of an overdose of amylene hydrate (tertiary-amylic-alcohol), a sedative drug which had been administered in error for a diluted preparation. It was stated at the inquest by the medical superintendent, Dr. John G. Porter Phillips, that the medical staff had been greatly overworked since the outbreak of war, owing to the absence of several members, including the second medical officer, the two house surgeons, and the steward, all of whom had been called up for active service. The result was, Dr. Phillips explained, that all the dispensing had to be performed by one of the medical officers. This officer, Dr. H. T. Jones, when called, stated that he acted as dispenser, and that on the day in question, when at work, he found the bottle which usually contained the dilute mixture empty. He therefore made up the draughts from another bottle, which he presumed to be a reserve stock, but which as a matter of fact contained the drug in concentrated form. The pathologist at St. Mary's Hospital, Dr. Bernard Spilsbury, who made an autopsy on each patient, stated that death was due to heart failure, consequent upon the administration of amylene hydrate. The jury returned a verdict in each case of "Death from misadventure."

### A DISPENSING REFORM IN MENTAL HOSPITALS.

But the most important part of Dr. Spilsbury's evidence, and the one which gives to this unfortunate occurrence its significance to the hospital world, is his remark that it was very desirable to have a regular dispenser in such an institution as Bethlem Hospital. It was better, in his opinion, to have a qualified chemist than a doctor; the chemist's methods were different. Even without the above evidence, and Dr. Spilsbury's emphasis, it certainly seems desirable for the governors of Bethlem to appoint a properly trained pharmacist to do the dispensing, for it is hardly just or reasonable to require the medical officers in large institutions to do all their own dispensing in addition to their other work. Even in His Majesty's prisons the prisoners have the services of a pharmacist at their disposal, and surely mental patients should have an equally good service? It is to be hoped that the Pharmaceutical Society and other organisations of workers in pharmacy will represent to the authorities the advantages of having a registered pharmacist in charge of the dispensary at all large institutions.

### THE NEW SANATORIUM FOR WOMEN.

The finance committee of the Sheffield City Council have approved the proposed expenditure on the scheme for the erection of a tuberculosis sanatorium for women in Rivelin Valley Road. The cost, exclusive of £2,750 for the purchase of the site, is £20,000. In the building accommodation would be provided for 100 patients and the necessary staff, and the plan is of a central block for acute cases and two detached convalescent pavilions. The central block would be designed to accommodate forty-six advanced patients, who would be treated in single- and double-bedded rooms, while the accommodation for the pavilion patients would consist of double-bedded rooms only. Each pavilion would be occupied by twenty-seven patients. Without the plans before us no detailed criticism can be given, but it is doubtful if any plan could reconcile an expert sanatorium or tuberculosis authority to double-bedded rooms, especially for advanced cases. Doubtless questions of cost and convenience have to be considered, but even an authority which proposed to build a sanatorium at about £200 per bed should not be tied to such an arrangement. Medical questions for the moment apart, all administrative experience shows that if you are to have more than one bed in one room, various numbers have advantages, except in the case of the number two, which has little in favour and much against its use. It was tried and has been given up in the best administered hospitals in the case of nurses' homes, and it is slowly but surely being given up in the parallel case of ward-maids' quarters. Why, therefore, should it be perpetuated in the latest sanatorium for women that is being built?

### FARM LABOUR FOR CONSUMPTIVES.

We are already familiar in this country with the graduated labour system in relation to treatment, and sporadic suggestions have also been made for turning suitable patients to such productive work as they may be capable of. But, broadly speaking, all these attempts have been confined to patients undergoing sanatorium treatment and have terminated on their discharge. News, however, comes from Australia of an attempt to make the open-air treatment not an episode of treatment but a habit of life, and the organisation suggested is that of a farm colony. Thus an Australian correspondent writes: "The oft-advocated farm and farm colony as a means of cure and also as a help towards the upkeep of tuberculous patients seems as if it might materialise shortly in Victoria. A scheme of the kind has been laid before the chief health officer, Dr. Robertson, by Dr. A. Brown, superintendent of the Green Vale Sanatorium for Consumptives. It is pleasant to learn that already at that institution suitable patients have the chance of learning much and practising something of rural industries, and Dr. Brown thinks that this kind of instruction and labour might be considerably extended. He advocates the establishment of a farm colony of not less than 5,000 acres, contiguous to Green Vale, that each patient should be given two or more acres to work, and debited with 10 per cent. of proceeds of sales of his produce in order to cover various items of cost. This, he thinks, would induce the sufferers to stay and continue sanatorium treatment, instead of returning to unsuitable surroundings—in the city mainly—as so many do, when only partially benefited."

### THE CRUX OF THE AFTER-CARE PROBLEM.

It is true that on November 1, 1913, we published the first of a series of articles by Dr. J. E. Esslemont on "Garden Cities for Consumptives," which embodied proposals for a national scheme of segregation. But, though the ideas were certainly stimulating, the scheme was cast on too large a scale to stand much chance of immediate inception. The Australian proposals, on the other hand, appear to aim rather at a constructive system of after-care, by providing an alternative of suitable surroundings, and with more humble intentions may, in the end, if adopted, prove far-reaching. At all events all engaged in the prevention and treatment of tuberculosis in this country are forcibly reminded by this scheme of two important things—one, the very haphazard attempts at after-care at present undertaken; another, the practical question whether any after-care system can be of much practical use which, content with only such supervision as dispensaries may be able to provide, fails to offer a definite continuance of the open-air life together with the possibility of the patients becoming largely self-supporting. We believe, at all events, that these two difficulties must be faced if our after-care endeavours are not to remain valueless, and, therefore, draw our readers' attention to the proposals of the Australian scheme.

### THE WOUNDED "KEEP THE HOSPITALS GOING."

An interesting discussion took place at the monthly meeting of the board of management of the King Edward VII.'s Hospital, Cardiff, in which the competing claims of wounded soldiers and of the civilian population for hospital treatment were sympathetically considered. It transpired that at present ninety-four of the beds of the hospital are placed at the disposal of the 3rd Western General Hospital, Cardiff, for the treatment of wounded soldiers, that there remained 185 beds at the disposal of the civilian population, and that no urgent case had been sent away. It was also contended that it would prove difficult to keep the hospital going in its entirety for civil cases, whilst many members of the honorary medical staff are absent on active service, and no fewer than eight are attached to the 3rd Western General Hospital. It is a fortunate coincidence that the military duties of the latter necessitate their daily presence at King Edward VII.'s Hospital. Ultimately a resolution was unanimously adopted that the use of the beds for the wounded soldiers should be continued. The decision appears to be advantageous to soldiers and civilians alike, having regard to the exceptional demands made under existing circumstances upon the honorary staffs of hospitals, accentuated, as they are, by the special difficulty of securing the services of resident house-surgeons.

### STATE AID FOR THE FINE CHEMICAL TRADE.

The decision of the Board of Trade to assist in the promotion of a company for the manufacture of fine chemicals is almost as important from the point of view of the manufacture of drugs as it is from that of the dye-stuffs industry. The shortage of aniline dyes and of a long list of drugs made under German patents has become so pronounced as a result of the war that the necessity for manufacturing products of these types has become urgent. But the industry is so complex and requires such a large amount of capital to establish on an economical scale that it is doubtful whether by private enterprise alone we should ever be able to compete with Germany. The use of duty-free alcohol is more essential to the production of synthetic drugs than to the manufacture of coal-tar colours, and it may almost be taken for granted that one of the first steps the Board of Trade will take in connection with the operation of the new scheme will be to secure the removal of any obstacle that stands in the way of the use of cheap manufacturing spirit. In the preparation of such substances as sulphonal, phenacetin, and antipyrin cheap alcohol will be absolutely necessary if British manufacturers are to compete successfully with their rivals abroad.

### TRIBUTE TO A HOSPITAL SECRETARY.

We have recorded in these columns as every occasion served the progress of the scheme of alterations and additions which is proceeding in connection with Addenbrooke's Hospital, Cambridge. Exactly how much work a scheme of this kind entails upon the hospital secretary in charge, all superintendents know only too well. It is therefore gratifying to see that the governors of Addenbrooke's Hospital have placed on record their appreciation of the services of Mr. R. J. Coles, the secretary-superintendent. In moving that a resolution be passed to this effect at the quarterly meeting, Mr. George Stace remarked that Mr. Coles had performed admirable work during the time when the institution had been in a state of chaos. He added, amid laughter, that he would not have been in Mr. Coles' shoes for double the amount of his salary. The secretary had been bandied from pillar to post, and had worked much harder than he ought to have done. These remarks are worth placing on record, not merely as indicating that class of high personal service for which the voluntary system is famous among paid and unpaid officials alike, but also as illustrating what a lately deceased and well-known administrator meant by remarking that he always regarded half his work as paid and half voluntary. While, therefore, we all know that each enthusiastic hospital manager does work which, from its skill and strenuousness, would receive far higher rewards according to commercial standards than are open to it in its own sphere at present, we must draw the moral, which is to pay the best men salaries worthy of their abilities so as to prevent them, in self-defence, from seeking another profession. Since you cannot be a first-rate hospital administrator without having made the spirit of personal service a part of yourself, there is no danger that under the voluntary system good men will only work in accordance with what is paid to them.

### ESSEX DOCTORS' GIFT TO THE WAR OFFICE.

The members of the medical profession in Essex have subscribed a sum of a little over £420, with which they have purchased a motor ambulance fully equipped with four stretchers and the necessary fittings, and have presented it to the War Office for the use of the wounded in France and Belgium. It is interesting to note that the subscriptions have been obtained by the various medical officers of health in the county, and a complete list of the donors can be seen at the office of the county medical officer of health, Dr. Thresh, at Chelmsford. The ambulance will bear a brass plate with the inscription: "This ambulance was presented to the War Office by the members of the medical profession in Essex, England, November 1914."

### THE WAR AND HOSPITAL SATURDAY.

It is good news, and a most satisfactory indication of the spirit apparently prevailing throughout the whole Hospital Saturday movement, that in Birmingham the contributions received up to date are practically the same as those recorded at the corresponding period of last year, with the result that there is a good chance of last year's grand total of £23,000 being passed. It would, however, be missing the point and value of the Saturday Fund movement to let common sense and patriotism alone account for this result. The hold on the affections of the people to-day of Hospital Saturday is the result of years of hard and enthusiastic work, and we are glad to notice that at the recent meeting of the Board of Delegates of the Birmingham Hospital Saturday Fund, Alderman Sir Hallewell Rogers emphasised how much the Fund's success has been due to the interest and energy of its honorary secretary, Alderman Aston. It is nineteen years since Mr. Aston was elected joint honorary secretary, and ever since 1899 he has occupied the position by himself, and has interested himself very actively in the varied schemes of the fund, which have sprung up since 1892, the year in which it ceased to be merely a collecting agency. As Sir Hallewell Rogers remarked, Mr. Aston has also taken upon himself the entire management and collection of the funds required for the Sir William Cook Memorial Home for Consumptives at Romsley Hill, to which allusion has recently been made in our columns. Sir Hallewell Rogers, on behalf of the executive committee, presented Mr. Aston with a bound address, containing illustrations of the Home and a short history of the movement. The patients at the Home are nearly all Hospital Saturday patients, and the occasion of the presentation was a most valuable reminder of the fact that the affection felt for the Saturday Fund movement to-day, and its many branches of work, are the best tribute to the quality of the men who have built up its reputation and usefulness. The state of the Saturday Fund to-day, in spite of the war, is the best memorial that Mr. Aston could desire.

### A PANEL DOCTOR "FINED."

A curious question of law, and expediency, was discussed at a meeting of the Derbyshire Insurance Committee last week, when Dr. J. Court raised the question of the legality of the action of the Medical Service Sub-Committee in fining a panel practitioner £5 for inequalities in granting certificates in respect of an insured person's inability to work. Dr. Court contended that the Sub-Committee's action was contrary to all principles of English law. The clerk, Mr. A. J. Ash, thereupon explained that the term "fine" was an incorrect one, and eventually the Committee, of which Mr. H. Fanshawe is chairman, decided that the sum should be demanded on the ground that it represented the amount which the Committee were entitled to assess as the expenses to which they had been put by reason of the panel practitioner's default.

### DISCIPLINE IN CONVALESCENT HOMES.

Some interesting points are made in the latest Report of the Red Cross Commissioner for the North-Eastern District of Scotland. Among those which are of more than local interest is one which deals with the problem of discipline in convalescent homes, which are now being used so much to take the lighter cases of wounded so as to leave hospital beds free for the urgent cases which continue to arrive from the Front. The Commissioner's Report points out that members of Voluntary Aid Detachments, being part of the technical reserve, are under the control of the military authorities, and that, in case of any disciplinary difficulty occurring, the medical officer should apply to the County Director for instructions as to how to proceed. Patients, it is added, should not be allowed greater freedom than in ordinary hospitals, and, as far as possible, when out for motor-drives and similar excursions, they should be attended by orderlies from the male Voluntary Aid Detachments. Cases have occurred in which soldiers have been allowed to enter public-houses, or have been treated when out of hospital, and precautions must be taken to prevent any such infringement of the ordinary hospital regulations. This is the natural and inevitable view, and is interesting to institutional officers as reminding them that problems of a similar nature are not exclusively confined to convalescent homes where wounded are seeking recovery, but that they have been the chief difficulty in such institutions, for instance, as sanatoria and tuberculosis hospitals. When the rush for superintendentships at sanatoria was at its height, consequent upon the passing of the Insurance Act, we pointed out again and again that special qualities were needed for these posts, and not only medical ones; but that a sanatorium administrator required almost as much a special training as a tuberculosis specialist. Now that our convalescent homes are filled with men on the high road to recovery, those responsible for these institutions are realising the problem to the full themselves.

### A DEATH AT A BOARD MEETING.

It is seldom that a member of any hospital board of management actually dies while attending a hospital meeting. Last week, however, Lieutenant-Colonel A. G. Holland passed away at the usual Wednesday house committee meeting, which is held at the Royal Hospital for Incurables, Putney Heath. Dr. S. Henning Belfrage, a member of the board, and Dr. John Gay (the medical officer of the institution), who were present, rendered prompt aid, which, however, was unavailing. Colonel Holland had been a member of the board of management of the Royal Hospital for Incurables for seventeen years. He was a distinguished member of a distinguished family; a man of great principles and strong opinions, who was regarded by his colleagues as a warm-hearted and sympathetic friend. Some thought that he had not enough patience with elementary economies, but if this were recognised as one of his traits, another of them was the way in which he entered into the work of the paid officials, so that, if their work or their systems happened to be faulty, he would identify himself with the blame. Colonel Holland, who was a brother of Canon Scott Holland, entered the Army in 1869, and was gazetted lieutenant-colonel in 1896. He retired in 1902. He served during the Afghan Campaign, and was present during the occupation of Kandahar, being mentioned in dispatches. He took part in the Boer War of 1881, and was a member of the Sudan Expedition of 1884-85, receiving the medal with clasp and the bronze star. He saw further service during the South African War, with the Imperial Yeomanry, and was given the Queen's medal with three clasps. On Saturday last the remains of the deceased gentleman were cremated at Golder's Green. Eight members of the board and three officials of the institution were present at the previous memorial service at Wimbledon.

### MEDICAL MEN ON PUBLIC BODIES.

The usefulness of medical men on public bodies is one on which we have often insisted, but the public, as a rule, does not realise how useful they can actually be when they have leisure to spare for public work. A pleasant instance of public recognition deserves to be recorded, however, in the case of Dr. W. S. Darby, who, as a member of the Harrow Urban District Council, has received the public thanks of the Council for "the able and efficient services he has rendered to the district by undertaking temporarily the duties of medical officer of health." We are glad to see not merely that this has been entered on the minutes, but also that one speaker, Mr. H. J. Strickland, drew the correct moral from the occasion—namely, that the way in which their interests had been looked after showed the wisdom of the public in electing a medical man to the Council. The speaker added that personally he hoped that there would always be a medical man upon it. Luckily, the value of medical men in public positions received its best possible testimony in the recent term of office of Sir Thomas Boor Crosby, who was, as we all remember, the first medical man to be elected Lord Mayor of London. But it is not only medical mayors who are wanted. The influence of practitioners on public bodies generally could only be advantageous, and we welcome every instance of its growth.

### BELGIAN SOLDIERS AS HOSPITAL COLLECTORS

The following incident is worth while recording. Thirty-three of the Belgian wounded soldiers have been discharged from the Torbay Hospital. The majority were sent direct to Folkestone for the Front. On leaving, each man was provided with a new pair of boots and a new set of undergarments. The point, however, is yet to come, for the twelve remaining Belgian soldiers were taken by motor-cyclists in side-cars to surrounding villages on Saturday last. They were thoughtfully provided with hospital collecting boxes. Result: £5 13s.

### WELSH DISSATISFACTION WITH SANATORIUM BENEFIT.

It would be hard to find a single body which has made itself in any way responsible for sanatorium benefit which has not felt the criticism that has fallen on this section of the Insurance Act in such full measure. A typical instance was again provided last week, when the Breconshire County Council adopted the report of a joint committee—representing the finance and the sanatorium benefit sub-committees—advising them against entering into a revised form of agreement, submitted by the Welsh National Memorial Association, on the ground that while rendering the Council liable for a proportion of the Association's net deficit (practically equal to a ½d. rate) it did not guarantee Breconshire patients sanatorium treatment. The County Medical Officer, Dr. Colston Williams, explained that in consequence of the delay in providing accommodation for certain cases before the County Insurance Committee, which were recommended for hospital treatment by the tuberculosis committee, a strongly worded complaint had been sent to the National Memorial Association. In the same report, moreover, payment of a 6d. rate on the 18,000 insured persons in the county to the panel doctors was criticised on the ground that from January 1913 to June 1914 only fifteen persons had been recommended for domiciliary treatment, and in the majority of these cases, it was added, the treatment was brief. The report also recommended the County Council to inform the Brecon Insurance Committee that if the Council were to undertake responsibility for any deficit on sanatoria benefit in future they would require the whole 1s. 3d. provided by the Insurance Act to be properly applied for that purpose. Dissatisfaction could hardly be more comprehensive, in spite of the Welsh National Memorial Association, the large amount of money spent, and the elaborate organisation.

### FIRST STEP IN THE ANTI-SYPHILIS CAMPAIGN.

The importance of refusing to allow all other questions to be swept aside or shelved owing to the war was well illustrated last week at the Royal Society of Medicine. On the occasion in question the National Council for Combating Venereal Diseases was formally inaugurated, and thus the first step was taken in the anti-syphilis campaign. Sir Thomas Barlow was in the chair, and the speakers in favour of the resolutions included such prominent personalities as the Dean of St. Paul's, Cardinal Bourne, and Lord Kinnaird, apart, of course, altogether from the medical speakers, among whom were Sir Rickman Godlee, Mr. Charters Symonds, and Sir Henry Morris. In spite of this, that meeting and those speeches were virtually boycotted by the lay Press, so little, apparently, do lay editors understand that the national life is not as a fact, and ought not on any sensible theory, to be brought to an end, even though military operations on a vast scale are actually in progress. As a matter of fact, from one point of view, the war has given to the National Council its opportunity, since the concentration of very large bodies of men in camps forces the problems involved on the consideration of both civil and military authorities. The Council's aims are to provide accurate and enlightened information on the prevalence of the disease and the need for its early treatment; to help to disseminate sound knowledge of the physiological laws of life; to co-operate with existing associations, and to arrange in connection with them courses of lectures and suitable pamphlets and literary matter. Lastly comes the aim "to promote such legislative, social, and administrative reforms as are relevant" to the above objects. It is on the interpretation of this last clause that the value of the National Council's work will depend. We trust that specific reforms for which it will work will be made known as soon as practicable, for though any definite programme is sure to encounter opposition, at the same time only a definite programme can enlist all the active forces on the Association's side under its banner.

### HOW TO HELP THE AMBULANCE SERVICE.

If they will only realise it the public will have a notable opportunity of helping the London ambulance service when it comes into operation in the near future. When an accident happens the ambulance is to be summoned by telephone, and it is therefore absolutely necessary that the nearest telephone shall be available. Many of the subscribers to the telephone system have already signified their willingness to allow the use of their instruments and the fixing of telephone indicators outside their premises. In some districts of London, however, the use of very many more instruments is desirable, and, indeed, it is hoped to secure the use of a telephone in every 440 yards of the more important thoroughfares. Any offers to co-operate will be gladly welcomed by the London County Council, and they will materially assist in the successful inauguration of a service which is necessary for London, and has been so long overdue. Now that it is almost an accomplished fact the public which criticised the delay in its formation can lend a hand to make it efficient in the way indicated above.

### BOOTS FOR THE BELGIAN WOUNDED.

We have already drawn attention to the need for boots for the Belgian wounded, which is urgently felt by hospitals anxious to discharge their convalescent patients to make room for new cases. Indeed, our information shows that men have actually been compelled to remain in hospital for want of this essential element of the equipment which they require on their discharge from the wards. Our request for information from every hospital where such need has arisen, and for subscriptions to provide a fund to supply boots—which, thanks to the co-operation of Messrs. Manfield and Son, of Northampton, can be supplied at 15s. 6d. a pair—has stimulated local interest in two important ways. Not only have subscriptions reached us, as we recorded last week, but several hospitals have started funds of their own, and been able to supply in some degree their own requirements thereby. Those who have adopted the latter course have fulfilled the spirit of our suggestion, if not the letter. Our business this week is therefore to urge the authorities of every hospital to make up their minds which procedure they will adopt. Decisions will depend on local circumstances. We hope they will be notified to us, when our object will soon be achieved. In response to our request in last week's issue we have to acknowledge with thanks the receipt of £1 11s. from M. E. H. for two pairs of boots.

### THIS WEEK'S DRUG MARKET.

The upward movement in the price of opium, and consequently of its alkaloids, has been accentuated during the past week, and the effect of the stoppage of shipments from Turkey seems likely to be more pronounced than was at first expected. The large supplies of Persian opium in London are not, it appears, adequate to prevent a rise in prices. Apparently no advantage is to be gained by delaying purchases to cover immediate requirements, as the possibility of a decline in value of this important drug is very remote. The advancing tendency in the price of carbolic acid is maintained. There is no substantial change in the position of quinine notwithstanding the belief that the Dutch Government is on the point of imposing an export duty; stocks of quinine in this country are large, but in course of time must be diminished considerably in view of the fact that new supplies are much curtailed. Gentian root is lower in price in consequence of new arrivals; digitalis leaves are also lower. A further reduction in quotations for citrates has followed the decline in value of citric acid. Santonin is getting scarcer and dearer. Ergot of rye is cheaper. On the whole business is steadily improving, and it is remarkable that the upward movement in prices is so slight.

